# Job Satisfaction among Ophthalmologists in Iran

**DOI:** 10.18502/jovr.v17i4.12315

**Published:** 2022-11-29

**Authors:** Hamideh Sabbaghi, Masomeh Kalantarion, Mehdi Yaseri, Bahareh Kheiri, Zhale Rajavi, Sare Safi

**Affiliations:** ^1^Ophthalmic Epidemiology Research Center, Research Institute for Ophthalmology and Vision Science, Shahid Beheshti University of Medical Sciences, Tehran, Iran; ^2^Department of Optometry, School of Rehabilitation, Shahid Beheshti University of Medical Sciences, Tehran, Iran; ^3^Department of Medical Education, Virtual School of Medical Education and Management, Shahid Beheshti University of Medical Sciences, Tehran, Iran; ^4^Ophthalmic Research Center, Research Institute for Ophthalmology and Vision Science, Shahid Beheshti University of Medical Sciences, Tehran, Iran; ^5^Department of Epidemiology and Biostatistics, School of Public Health, Tehran University of Medical Sciences, Tehran, Iran; ^6^Negah Specialty Ophthalmic Research Center, Shahid Beheshti University of Medical Sciences, Tehran, Iran; ^7^Department of Ophthalmology, School of Medicine, Shahid Beheshti University of Medical Sciences, Tehran, Iran; ^9^https://orcid.org/0000-0002-2627-7222; ^10^https://orcid.org/0000-0003-4778-3973

**Keywords:** Associated Factors, Iran, Job Satisfaction, Ophthalmologists, Warr-Cook-Wall Questionnaire

## Abstract

**Purpose:**

To estimate the level of job satisfaction among ophthalmologists in Iran and determine the associated factors that may impact their overall job satisfaction.

**Methods:**

In this cross-sectional study, 181 ophthalmologists (79.0% male) were interviewed by the Warr-Cook-Wall questionnaire with 7-point-Likert scale, which transformed responses from a 0 (most dissatisfied) to 100 (most satisfied). Questionnaires were randomly distributed among registered ophthalmologists at the 29 ${}^{th}$ Annual Congress of the Iranian Society of Ophthalmology in November 2019. Satisfaction under each scale was considered as a score of > 60% of the total score.

**Results:**

A total of 181 ophthalmologists with a mean age of 47.8 ± 12.1 years and 16 ± 12 years of practice participated in the present study. They were mostly satisfied with their job as a whole (88.1%, mean score: 60.6 ± 20.7) and dissatisfied with their income (55.9%, mean score: 47.6 ± 20.3). High levels of job satisfaction was found among ophthalmologists with longer duration of practice (P < 0.001) while lower levels of satisfaction were identified among those who worked in academic centers (P = 0.004).

**Conclusion:**

In this study,high levels of job satisfaction were found among ophthalmologists with longer duration of practice while low levels of satisfaction were identified among ophthalmologists who worked in academic centers. The factors of salary and working hours accounted for the least levels of job satisfaction.

##  INTRODUCTION

Job satisfaction refers to the level of contentment that a person feels toward their job which is influenced by their expectations and how that feeling translates into their attitude and achievements.^[1,2,3]^ Job satisfaction is entirely obtainable in cases where successful achievements were matched with expectations, desires, and values in the profession.^[2,3]^

In general, job satisfaction which can be influential on different aspects of an occupation^[4]^ is reported to have a positive relationship with creativity and productivity, the quality of work, the provision of higher work incentives, and lower absenteeism rates.^[5]^In addition, lack of attention to employees' satisfaction can threaten the financial progression of each organization and may eventually lead to a gradual economic decline. Job dissatisfaction may also cause anxiety, lower productivity, absenteeism, resignation, job abandonment, early retirement, as well as individual physical and mental discomforts.^[6,7,8,9]^

Job satisfaction of healthcare providers, especially physicians, is a substantial requirement in the healthcare system which can have a crucial role in improving physicians' performance and, ultimately, the stability of the healthcare system,^[10,11,12]^ while job dissatisfaction may lead to reduced patient care by physicians and increased healthcare system costs.^[12,13]^ A significant association between physicians' job satisfaction and patients' satisfaction was reported by some other studies.^[14,15]^ Furthermore, job satisfaction is also an influential factor affecting the mental health of physicians and other health caregivers.^[16]^

It is reported that organizational and environmental factors impact job satisfaction either directly or indirectly.^[7]^ Other factors that affect job satisfaction include salary and benefits, job security, justice, non-discrimination among employees, and access to work equipment.^[17]^

A study conducted in Norway showed that most psychiatrists and primary care physicians have higher levels of job satisfaction as compared to other medical groups.^[18]^ Furthermore, higher levels of job satisfaction were reported among Norwegian physicians as compared to German physicians, which was due mainly to appropriate working hours, adequate salary, and good control over work affairs.^[19]^ Conversely, some other studies showed lower levels of job satisfaction among physicians who work in the United States and the United Kingdom.^[16,20]^

As a consequence, identification of and addressing the known factors affecting job satisfaction is a fundamental step in improving productivity and quality of the job. Although several studies exist investigating job satisfaction among other professions in Iran,^[3,9]^ none was conducted among ophthalmologists. Therefore, we aimed to estimate ophthalmologists' job satisfaction levels in Iran and determine the associated factors that may impact it.

##  METHODS

The present cross-sectional study investigated the job satisfaction levels among 181 Iranian ophthalmologists (79.0% male) who participated in the 29 ${}^{th}$ Annual Congress of the Iranian Society of Ophthalmology in November 2019.

This study was approved by the Ethics Committee of the Ophthalmic Research Center affiliated to Shahid Behshti University of Medical Sciences, Tehran, Iran via the approval code IR.SBMU.ORC.REC.1394.04. The study details were explained to all participants and their information was kept confidential.

### 10-item Warr-Cook-Wall Questionnaire 

Data collection was done using the 10-item Warr-Cook-Wall questionnaire which was customized and translated into the Persian language by Lavasani et al.^[21,22]^ This questionnaire was presented to an expert panel including five ophthalmologists (three academic and two non-academic ophthalmologists) to revise it so that it would be aligned with the ophthalmology context. The revised questionnaire was then approved by other professors at the department of ophthalmology.

All participants were interviewed by the10-item Warr-Cook-Wall questionnaire consisting of 10 questions scored based on a 7-point Likert scale. This questionnaire recorded general information regarding the interviewees' age, sex, location of practice, field and duration of practice, and their subspecialty. The job satisfaction of all participants was also recorded according to their responsibility, freedom to choose how to work, variety of work, relationship with colleagues, feelings toward the job, physical condition, opportunities, salary, acknowledgement of good performance, and working hours. For data analysis, the responses under each of the 10 items were transformed into a 0 (most dissatisfied) to 100 (most satisfied) scale.

The questionnaire was filled out by the study participants at the time of registration for the Congress in order to prevent duplication.^[23,24]^ In order to increase the response rate, participants were gifted a Reduced Snellen Eye chart.

**Table 1:** Demographic characteristics of the study subjects.


** Factors**	** Level**		**Value **
Age (yrs)	Mean ± SD		47.8 ± 12.1
	Median (IQR)		47 (37 to 59)
Age category (yrs)	≤ 35		34 (18.8%)
	36–50		76 (42.0%)
	51–65		60 (33.1%)
	≥ 66		11 (6.1%)
Sex (%)	Male		143 (79.0%)
	Female		38 (21.0%)
Duration of practice (yrs)	Mean ± SD		16 ± 12
	Median (IQR)	14 (5 to 23)
	≤ 10	78 (43.1%)
	11–25	68 (37.6%)
	≥ 26	35 (19.3%)
Last degree (%)	General ophthalmologist	115 (63.5%)
	Fellowship	66 (36.5%)
Field of fellowship (%)	Anterior segment	27 (40.9%)
	Posterior segment	23 (34.8%)
	Strabismus	12 (18.2%)
	Glaucoma	4 (6.1%)
Faculty (%)	Research	6 (3.3%)
	Educational	35 (19.3%)
	Non-academic	140 (77.3%)
Status (%)	Private office	124 (68.5%)
	Academic staff	57 (31.5%)
	
	
IQR, inter quartile range; SD, standard deviation; yrs, years				

**Table 2:** Mean job satisfaction scores of study participants for every 10 questions of the questionnaire.


**Items**	**Satisfaction Score**		**Satisfied**
	**Mean ± SD**	**Median (IQR)**	***N*** ** (%)**
Responsibility	58 ± 17.3	50 (50 to 66.7)	52(88.1%)
Freedom to choose how to work	61 ± 19.2	66.7 (50 to 66.7)	58 (98.3%)
Variety of work	61.2 ± 18.9	66.7 (50 to 66.7)	55 (93.2%)
Colleague	55.5 ± 18	50 (50 to 66.7)	47(79.7%)
Feeling of satisfaction	60.6 ± 20.7	66.7 (50 to 66.7)	52(88.1%)
Physical condition	57.6 ± 20.1	50 (50 to 66.7)	49(83.1%)
Opportunity	54.7 ± 16.9	50 (50 to 66.7)	39(67.2%)
Salary	47.6 ± 20.3	50 (33.3 to 50)	33(55.9%)
Acknowledgement of good performance	49.2 ± 21.2	50 (33.3 to 66.7)	39(66.1%)
Working hours	52 ± 19	50 (50 to 66.7)	36(61%)
	
	
IQR, inter quartile range; SD, standard deviation			

**Table 3:** Job satisfaction of participants based on their characteristics.


** Factors**	**Level**	**Satisfied**	***P*** **-value**		**AOR**	<@orange**95% CI**		***P*** **-value**		**AOR (backward)**	<@orange**95% CI**		***P*** **-value**
				**Lower**	**Upper**		**Lower**	**Upper**	
Age (yrs)	≤ 35	4 (11.8%)	0.001	R				
	36–50	24 (32.0%)		2.501	0.7	8.931	0.158			
	51–65	23 (38.3%)		1.839	0.369	9.169	0.457			
	≥ 66	8 (72.7%)		3.007	0.31	29.15	0.342			
								
Sex (%)	Male	52 (36.4%)	0.05	R			R		
	Female	7 (18.9%)		0.421	0.16	1.109	0.08	0.425	0.164	1.103	0.079
								
Duration of practice (yrs)	≤ 10	16 (20.5%)	< 0.001	R			R		
	11–25	22 (32.8%)		1.347	0.509	3.565	0.549	1.602	0.737	3.483	0.234
	≥ 26	21 (60.0%)		4.634	1.075	19.974	0.04	5.472	2.202	13.599	< 0.001
								
Field of fellowship (%)	Anterior segment	10 (37.0%)	0.807					
	Posterior segment	9 (39.1%)						
	Strabismus	6 (50.0%)						
	Glaucoma	1 (25.0%)						
								
Status (%)	Private office	43 (36.8%)	0.004	R			R		
	Academic staff	16 (25.4%)		0.322	0.139	0.746	0.008	0.299	0.131	0.679	0.004
								
Faculty (%)	Research	1 (16.7%)	0.282					
	Educational	15 (42.9%)						
	Non- academic	43 (30.9%)						

white<bcol>16</ecol> ${}^{§}$ *P*-value for the comparison of the level to the reference level. *These variables remained statistically significant in the final model based on logistic regression. R, reference category.

**Figure 1:**
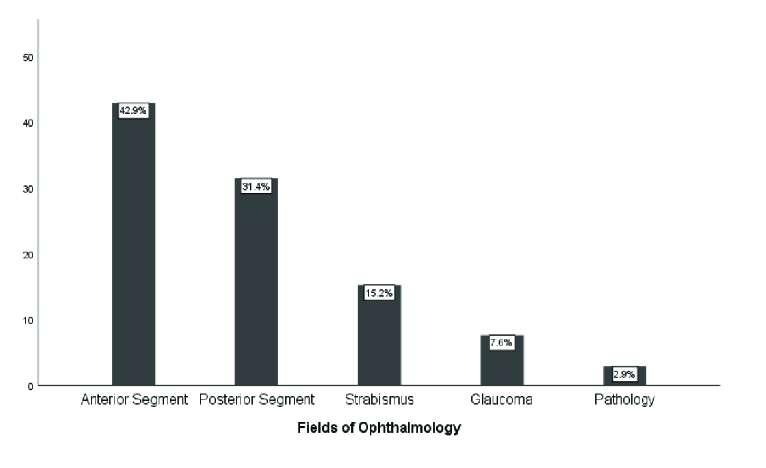
The frequency of job satisfaction in different fields of ophthalmology.

### Statistical Analysis

To present data, we used mean, standard deviation, median, range, frequency, and percentage analyses. To identify the associated factors of job satisfaction, all items of the questionnaire were first entered into the univariate model. Each variable with a *P*-value < 0.2 was statistically acceptable in order to enter the multivariate analysis. All statistical analyses were performed using the SPSS software version 25 (IBM Corp. Armonk, NY: IBM Corp.). *P*-values < 0.05 were considered statistically significant.

##  RESULTS

A total of 181 ophthalmologists with the mean age of 47.8 ± 12.1 years participated in the present study. Table 1 shows the demographic characteristics, academic degrees, duration and location of practice. The majority of them (63.5%) were general ophthalmologists and 56.9% had a duration of practice of > 10 years. A total of 22.6% of participants were faculty members of whom 3.3% and 19.3% were in the fields of research and education, respectively.

Table 2 shows the mean score of the individuals' job satisfaction in each item of the Warr-Cook-Wall questionnaire. A high level of satisfaction was observed in more than half of the study subjects (63.5%). Highest job satisfaction levels were positively related to the following factors: freedom to choose how to work (98.3%); variety of work (93.2%); level of responsibility (88.1%); and feelings of satisfaction (88.1%). The lowest levels of job satisfaction were identified in the following categories: acknowledgement of good performance (66.1%); working hours (61.0%); and salary (55.9%).

Table 3 shows the level of job satisfaction in relation to the characteristics of the participants. We found that higher levels of job satisfaction were observed among older ophthalmologists (*P* = 0.001), who had longer duration of practice (*P* < 0.001) and worked in a private office (*P* = 0.004). The simultaneous analysis also showed that ophthalmologists with longer years of experience were more satisfied as compared to those having < 10 years of experience (AOR = 5.472, 95% CI: 2.202–13.599; *P* < 0.001). Furthermore, ophthalmologists working in academic centers had lower levels of job satisfaction than those who worked in private offices (AOR = 0.299, 95% CI: 0.131–0.679; *P* = 0.004).

Figure 1 shows the frequency of job satisfaction in different fields of ophthalmology. As seen, the highest (40.9%) and the lowest (6.1%) levels of satisfaction were observed among ophthalmologists working in the fields of anterior segment and glaucoma, respectively.

##  DISCUSSION

The main aim of the present study was to determine the job satisfaction levels among Iranian ophthalmologists. In this regard, the Persian version of the 10-item Warr-Cook-Wall questionnaire was filled by Iranian ophthalmologists who participated in the 29 ${}^{th}$ Annual Congress of the Iranian Society of Ophthalmology.

The present study demonstrated that more than half of the ophthalmologists had a high level of job satisfaction. However, the highest and lowest levels were related to the freedom to choose how to work and salary, respectively. The highest levels of job satisfaction were significantly observed among ophthalmologists who were older and those who had longer years of practice. Additionally, lower levels of satisfaction were identified among ophthalmologists who worked in academic centers.

Our literature review shows that although most studies investigated job satisfaction among medical professionals, especially nurses,^[25,26,27]^ few studies focused on ophthalmologists.^[28,29]^ In this regard, the discoveries of a study conducted by Nair et al to investigate the job satisfaction among Indian ophthalmologists can be used for comparison with the study on Iranian ophthalmologists.^[28]^

Overall, the present study showed an acceptable level of job satisfaction (63.5%) among Iranian ophthalmologists, which is consistent with the results reported among Indian and Nigerian ophthalmologists with job satisfaction levels of 54.21% and 78.5%, respectively.^[28,29]^ Additionally, mean levels of satisfaction were reported among optometrists (145.9 ± 14.44) as compared to other medical specialists.^[30]^ Furthermore, high levels of job satisfaction were identified among 37.5% of the vision technicians studied by Paudel et al.^[31]^

Of note, multiple style questionnaires were also used to assess ophthalmologists' job satisfaction in related studies.^[28,29][31]^ In this investigation, the 10-item Warr-Cook-Wall questionnaire was used,^[21]^ while in other studies, a questionnaire created by a researcher was used for data collection, whose responses were provided based on literature review and panel experts' consensus.^[28,29][31]^

A standard questionnaire (job satisfaction survey scale) designed by Paul E Spector for optometrists was applied in the study by Chen et al.^[30]^ This questionnaire also assesses job satisfaction from different dimensions including promotion, supervision, operating conditions, relationships with coworkers, work nature, and communication.^[30]^

In the present study, the highest level of job satisfaction was related to the freedom to choose how to work (98.3%). This can be attributed to the fact that the majority of our study population who were general ophthalmologists (63.5%) and also worked in private office (68.5%) had the freedom to choose how to work. However, the study by Paudel et al^[31]^ reported acceptable levels of job satisfaction with the variety of work (70.8%) among vision technicians which is not in line with our findings. In some other studies, it was also reported that the highest levels of satisfaction were observed under the categories of patients' treatment (94.9%) and the nature of the work.^[29,30]^

In the present study, the lowest level of job satisfaction was related to salary (55.9%), since the majority of our population had long practicing experience and they expected to earn higher levels of income. The highest level of job dissatisfaction (71.2%) among Nigerian ophthalmologists was related to work facilities.^[29]^ The results reported by other studies on ophthalmologists,^[29]^ vision technicians,^[31]^ doctors working at teaching hospitals,^[32]^ and medical officers^[33]^ indicated the lowest levels for salary satisfaction. However, the results of the study conducted on optometrists in Malaysia demonstrated moderate salary satisfaction.^[30]^

Various studies reported a high level of satisfaction with colleagues, the rate of 78.7% was reported among Nigerian ophthalmologists^[29]^ and 66.7% among vision technicians.^[31]^

A low level of satisfaction with working hours (61.0%) was determined in the present study, which is consistent with the results reported on Indian ophthalmologists.^[28]^

In this study, there were higher levels of job satisfaction among ophthalmologists with older ages (*P* = 0.001) and long duration of practice (*P* < 0.001); however, Paudel et al found no significant relationship between job satisfaction and age and duration of practice.^[31]^ Although the association between job satisfaction and all of the underlying factors was not examined in the study on the Indian ophthalmologists, more challenges were reported among female ophthalmologists (*P* < 0.001).^[28]^

Meanwhile, Jain et al found that female ophthalmologists complained about having fewer working hours and experiencing extra obstacles regarding job promotions and discrimination in the workplace. However, no evaluation of the job satisfaction was performed between genders in the current study.^[34]^

Multivariate analysis shows high levels of satisfaction among ophthalmologists with longer experience ( < 0.001) and lower levels of satisfaction among those working in academic centers (*P* = 0.004).

It is noticeable that the lowest levels of job satisfaction recorded among the Iranian ophthalmologists in this study were regarding their salary, acknowledgement of good performance, and working hours. It is necessary for these concerns to be investigated fully by the health policymakers and the Iranian Society of Ophthalmology since high levels of job satisfaction can have a crucial impact when offering high-quality medical and educational services. Extensive investigation using a combination of qualitative and quantitative research measures on a larger ophthalmologists' population is recommended for future studies on job satisfaction.

One advantage of the present study was the usage of the Persian version of the Warr-Cook-Wall questionnaire^[22]^ which enhanced the efficiency and comprehensibility of this questionnaire. Sample selection from the ophthalmology congress participants can be taken as a possible limitation of the present study, since it is expected that congress attendees are already more satisfied in their jobs and their responses may skew the results. Additionally, the higher male respondent among our study participants could be considered as another limitation of the current study as it may not be accurately representative of the proportion of the male versus female ophthalmologists in Iran.

In summary, high levels of job satisfaction were found among ophthalmologists with longer duration of practice, while low levels of job satisfaction were identified among ophthalmologists who worked in academic centers. Salary and working hours were determined to be the factors that were responsible for the lowest levels of job satisfaction assessed in this study.

##  Financial Support and Sponsorship

None.

##  Acknowledgements 

The authors would like to express their gratitude to the Ophthalmology Research Center, Shahid Beheshti University of Medical Sciences and all the loved ones who cooperated with this research project, especially ophthalmologists.

##  Conflicts of Interest

The authors declare that they have no conflict of interest.
